# Supplementary material for: A Likelihood Approach for Real-Time Calibration of Stochastic Compartmental Epidemic Models
Source: PLoS Comput Biol. 2017 Jan 17;13(1):e1005257. doi: 10.1371/journal.pcbi.1005257 (PMC5240920; doi:10.1371/journal.pcbi.1005257)
Supplement: S1 File — (TAR.GZ) [file pcbi.1005257.s014.tar.gz › HSPH_Online-SI-Revision/IPoi09/n10-mild/n10-mild_BENCH2_table8.pdf]

1	newly 3 weeks spec	60.	179.153	{49., 402.}	205.989	0.400284	newly 3 weeks cumul	239.	632.78	{237., 1256.}	169.286	0.360464	newly 5 weeks spec	59.	125.525	{29., 325.}	135.967	0.309025	newly 5 weeks cumul	350.	910.356	{310., 1855.}	167.182	0.351417	newly 42 weeks cumul	920.	1466.44	{378., 3661.}	94.4252	0.272481
2	newly 3 weeks spec	35.	72.2692	{25., 154.}	120.989	0.278956	newly 3 weeks cumul	125.	313.25	{128., 712.}	154.631	0.320098	newly 5 weeks spec	13.	37.1923	{13., 79.}	192.308	0.376312	newly 5 weeks cumul	172.	402.327	{160., 859.}	139.747	0.29917	newly 47 weeks cumul	189.	486.173	{183., 1000.}	161.345	0.330828
3	newly 3 weeks spec	49.	56.0556	{15., 97.}	87.9441	0.334945	newly 3 weeks cumul	214.	266.259	{71., 430.}	86.6736	0.296026	newly 5 weeks spec	21.	25.1481	{6., 42.}	89.5944	0.33917	newly 5 weeks cumul	265.	329.481	{89., 541.}	87.2257	0.297794	newly 50 weeks cumul	281.	377.87	{105., 636.}	91.6766	0.286159
4	newly 3 weeks spec	42.	137.877	{44., 203.}	229.198	0.403637	newly 3 weeks cumul	187.	534.	{187., 865.}	187.363	0.372746	newly 5 weeks spec	18.	84.5614	{27., 128.}	369.981	0.519471	newly 5 weeks cumul	235.	727.333	{243., 1154.}	210.37	0.391321	newly 45 weeks cumul	289.	967.754	{303., 1498.}	235.604	0.409211
5	newly 3 weeks spec	82.	123.514	{41., 241.}	76.8622	0.24879	newly 3 weeks cumul	270.	415.27	{130., 811.}	76.0661	0.241097	newly 5 weeks spec	96.	103.595	{30., 222.}	55.9403	0.248573	newly 5 weeks cumul	451.	635.541	{190., 1272.}	69.9587	0.239273	newly 25 weeks cumul	1188.	1314.78	{378., 2783.}	58.4835	0.252008
6	newly 3 weeks spec	58.	16.6735	{4., 40.}	73.2231	0.748532	newly 3 weeks cumul	223.	86.2653	{16., 190.}	68.1248	0.583276	newly 5 weeks spec	18.	6.06122	{0., 20.}	72.6757	0.895981	newly 5 weeks cumul	288.	102.204	{19., 240.}	69.4728	0.621126	newly 51 weeks cumul	298.	110.061	{20., 280.}	68.8467	0.609353
7	newly 3 weeks spec	72.	46.9318	{14., 90.}	63.4785	0.390006	newly 3 weeks cumul	231.	217.068	{70., 399.}	60.9111	0.285302	newly 5 weeks spec	20.	21.0909	{4., 46.}	75.4545	0.351558	newly 5 weeks cumul	292.	270.432	{89., 514.}	61.3714	0.293017	newly 47 weeks cumul	336.	312.932	{101., 604.}	62.0198	0.295637
8	newly 3 weeks spec	137.	149.148	{58., 301.}	51.1547	0.215098	newly 3 weeks cumul	446.	560.623	{231., 1138.}	54.5468	0.197678	newly 5 weeks spec	94.	94.3607	{32., 190.}	54.0635	0.248877	newly 5 weeks cumul	661.	771.934	{316., 1555.}	52.6252	0.205443	newly 42 weeks cumul	1278.	1105.57	{486., 2273.}	47.4845	0.247459
9	newly 3 weeks spec	36.	146.873	{55., 243.}	307.98	0.520093	newly 3 weeks cumul	178.	569.818	{241., 1031.}	220.633	0.436791	newly 5 weeks spec	6.	88.3818	{29., 142.}	1373.03	1.04126	newly 5 weeks cumul	197.	771.473	{310., 1341.}	291.611	0.510644	newly 46 weeks cumul	197.	1037.35	{389., 1779.}	426.571	0.622181
10	newly 3 weeks spec	48.	64.3947	{23., 108.}	75.1645	0.241832	newly 3 weeks cumul	183.	257.447	{99., 507.}	74.7915	0.227373	newly 5 weeks spec	54.	34.1053	{8., 61.}	66.9591	0.427302	newly 5 weeks cumul	294.	339.316	{129., 644.}	66.1117	0.246667	newly 43 weeks cumul	610.	433.474	{173., 824.}	62.2347	0.36207
11	newly 3 weeks spec	38.	23.1579	{8., 46.}	45.4294	0.304291	newly 3 weeks cumul	143.	110.895	{52., 186.}	38.6456	0.225169	newly 5 weeks spec	2.	9.5	{3., 18.}	380.263	0.625209	newly 5 weeks cumul	158.	135.211	{66., 226.}	38.8741	0.209783	newly 48 weeks cumul	209.	152.684	{70., 253.}	37.1695	0.231507
12	newly 3 weeks spec	4.	26.0566	{6., 46.}	553.302	0.616558	newly 3 weeks cumul	21.	132.377	{32., 236.}	530.368	0.61843	newly 5 weeks spec	0.1	9.75472	{0., 24.}	9677.36	1.61395	newly 5 weeks cumul	21.	157.679	{36., 285.}	650.854	0.693941	newly 50 weeks cumul	21.	172.642	{41., 311.}	722.102	0.736972
13	newly 3 weeks spec	14.	214.892	{85., 449.}	1434.95	1.08754	newly 3 weeks cumul	103.	765.492	{319., 1629.}	643.196	0.791947	newly 5 weeks spec	15.	152.015	{49., 354.}	913.436	0.8724	newly 5 weeks cumul	131.	1098.89	{438., 2406.}	738.849	0.833414	newly 42 weeks cumul	149.	1709.95	{580., 3830.}	1047.62	0.946715
14	newly 3 weeks spec	78.	141.246	{45., 259.}	99.8619	0.253771	newly 3 weeks cumul	298.	519.062	{196., 966.}	90.5576	0.239825	newly 5 weeks spec	48.	95.8923	{28., 148.}	121.186	0.263575	newly 5 weeks cumul	412.	733.4	{254., 1309.}	95.9709	0.244529	newly 43 weeks cumul	587.	1095.	{344., 1910.}	106.225	0.248121
15	newly 3 weeks spec	142.	82.625	{33., 151.}	48.6444	0.336465	newly 3 weeks cumul	509.	332.725	{129., 610.}	45.4371	0.290764	newly 5 weeks spec	97.	46.475	{14., 103.}	55.4897	0.433756	newly 5 weeks cumul	755.	440.125	{173., 853.}	48.7781	0.33287	newly 45 weeks cumul	1453.	571.65	{204., 1177.}	60.6573	0.479999
16	newly 3 weeks spec	54.	111.476	{48., 179.}	114.256	0.276776	newly 3 weeks cumul	234.	465.841	{196., 724.}	106.756	0.270113	newly 5 weeks spec	35.	60.5714	{24., 101.}	91.746	0.252503	newly 5 weeks cumul	327.	610.619	{255., 935.}	96.4808	0.25101	newly 46 weeks cumul	351.	778.524	{314., 1191.}	127.88	0.300432
17	newly 3 weeks spec	90.	265.158	{161., 389.}	195.029	0.447757	newly 3 weeks cumul	301.	856.842	{590., 1155.}	184.665	0.438149	newly 5 weeks spec	86.	211.684	{119., 316.}	147.001	0.361422	newly 5 weeks cumul	488.	1312.79	{855., 1818.}	169.014	0.410615	newly 43 weeks cumul	2262.	2496.24	{1276., 4086.}	39.2212	0.164442
18	newly 3 weeks spec	41.	146.288	{43., 334.}	259.522	0.432232	newly 3 weeks cumul	174.	524.808	{176., 1202.}	204.377	0.387172	newly 5 weeks spec	45.	92.4038	{22., 226.}	134.829	0.311973	newly 5 weeks cumul	264.	732.923	{234., 1689.}	183.77	0.355723	newly 43 weeks cumul	314.	1063.85	{313., 2441.}	242.026	0.40108
19	newly 3 weeks spec	42.	32.7547	{11., 61.}	51.0332	0.29048	newly 3 weeks cumul	145.	151.34	{60., 263.}	53.1425	0.227416	newly 5 weeks spec	27.	16.7547	{5., 34.}	54.4375	0.371403	newly 5 weeks cumul	208.	191.132	{76., 324.}	50.3628	0.24597	newly 46 weeks cumul	266.	226.151	{84., 396.}	50.5178	0.265543
20	newly 3 weeks spec	94.	104.246	{33., 188.}	60.1717	0.240003	newly 3 weeks cumul	348.	447.842	{159., 796.}	65.5223	0.22731	newly 5 weeks spec	48.	55.0702	{17., 107.}	64.0716	0.248507	newly 5 weeks cumul	453.	578.456	{197., 1048.}	65.9347	0.230278	newly 47 weeks cumul	551.	716.544	{243., 1265.}	68.0358	0.23213
21	newly 3 weeks spec	33.	72.2692	{25., 154.}	130.653	0.288799	newly 3 weeks cumul	145.	313.25	{128., 712.}	123.886	0.275867	newly 5 weeks spec	16.	37.1923	{13., 79.}	143.269	0.313882	newly 5 weeks cumul	184.	402.327	{160., 859.}	126.599	0.281044	newly 47 weeks cumul	205.	486.173	{183., 1000.}	143.349	0.306395
22	newly 3 weeks spec	79.	111.766	{50., 217.}	62.8602	0.19382	newly 3 weeks cumul	247.	450.872	{226., 843.}	90.6021	0.236853	newly 5 weeks spec	60.	67.0638	{23., 138.}	56.1702	0.226883	newly 5 weeks cumul	392.	606.34	{289., 1149.}	71.548	0.208485	newly 43 weeks cumul	671.	817.426	{333., 1654.}	55.0908	0.201048
23	newly 3 weeks spec	1.	59.6222	{15., 70.}	5862.22	1.54165	newly 3 weeks cumul	20.	257.489	{70., 286.}	1187.44	0.899399	newly 5 weeks spec	0.1	25.5556	{5., 28.}	25 455.6	2.12237	newly 5 weeks cumul	20.	322.467	{86., 359.}	1512.33	0.988223	newly 48 weeks cumul	20.	375.311	{93., 435.}	1776.56	1.04809
24	newly 3 weeks spec	31.	32.9211	{10., 66.}	64.7708	0.293215	newly 3 weeks cumul	126.	143.079	{47., 278.}	59.7953	0.236389	newly 5 weeks spec	21.	17.1316	{2., 34.}	67.0426	0.438576	newly 5 weeks cumul	167.	185.263	{59., 352.}	60.9833	0.246729	newly 44 weeks cumul	190.	235.605	{70., 463.}	69.0443	0.252759
25	newly 3 weeks spec	26.	44.9487	{11., 99.}	103.057	0.283778	newly 3 weeks cumul	124.	224.949	{71., 565.}	103.371	0.263738	newly 5 weeks spec	3.	17.4103	{1., 42.}	499.145	0.670324	newly 5 weeks cumul	138.	270.564	{83., 668.}	115.645	0.276474	newly 51 weeks cumul	140.	300.897	{90., 772.}	130.531	0.289906
26	newly 3 weeks spec	39.	34.1053	{8., 61.}	64.6424	0.324794	newly 3 weeks cumul	98.	146.263	{45., 258.}	85.1772	0.242271	newly 5 weeks spec	10.	18.5526	{4., 39.}	118.684	0.334074	newly 5 weeks cumul	122.	190.579	{68., 347.}	89.4305	0.241856	newly 42 weeks cumul	155.	240.421	{79., 438.}	91.7487	0.251952
27	newly 3 weeks spec	6.	115.83	{45., 220.}	1830.5	1.18887	newly 3 weeks cumul	37.	460.396	{189., 842.}	1144.31	1.00891	newly 5 weeks spec	1.	65.4151	{18., 118.}	6441.51	1.68958	newly 5 weeks cumul	40.	611.66	{248., 1117.}	1429.15	1.0929	newly 44 weeks cumul	46.	809.736	{315., 1575.}	1660.3	1.14725
28	newly 3 weeks spec	58.	71.6486	{20., 142.}	68.5461	0.267352	newly 3 weeks cumul	196.	247.432	{70., 539.}	66.8092	0.253438	newly 5 weeks spec	40.	54.5405	{13., 116.}	75.4054	0.263528	newly 5 weeks cumul	279.	366.541	{97., 804.}	70.8999	0.258791	newly 22 weeks cumul	839.	689.135	{181., 1503.}	56.6794	0.322212
29	newly 3 weeks spec	51.	87.0794	{29., 137.}	90.5384	0.239275	newly 3 weeks cumul	209.	359.81	{125., 537.}	88.3193	0.230614	newly 5 weeks spec	29.	45.7302	{16., 71.}	83.1965	0.231498	newly 5 weeks cumul	281.	470.444	{170., 694.}	84.9291	0.224227	newly 45 weeks cumul	388.	591.508	{209., 888.}	76.7509	0.217008
30	newly 3 weeks spec	44.	72.2459	{23., 119.}	95.0447	0.255064	newly 3 weeks cumul	129.	298.361	{104., 460.}	140.869	0.287903	newly 5 weeks spec	32.	41.4262	{12., 64.}	75.9734	0.26634	newly 5 weeks cumul	194.	393.557	{130., 603.}	118.396	0.261832	newly 44 weeks cumul	268.	514.475	{164., 769.}	111.127	0.254667
31	newly 3 weeks spec	39.	20.725	{2., 43.}	70.9615	0.629602	newly 3 weeks cumul	120.	113.725	{21., 255.}	76.2292	0.404677	newly 5 weeks spec	8.	7.275	{0., 23.}	95.9375	0.773655	newly 5 weeks cumul	150.	133.05	{24., 304.}	76.1333	0.432866	newly 51 weeks cumul	165.	142.6	{25., 323.}	76.4848	0.447035
32	newly 3 weeks spec	110.	103.595	{30., 222.}	52.1622	0.264013	newly 3 weeks cumul	385.	343.784	{101., 714.}	49.6525	0.263031	newly 5 weeks spec	87.	83.027	{22., 190.}	57.4402	0.288393	newly 5 weeks cumul	596.	519.568	{146., 1111.}	50.2449	0.271866	newly 24 weeks cumul	1460.	1028.27	{284., 2210.}	52.8286	0.336719
33	newly 3 weeks spec	26.	137.877	{44., 203.}	430.297	0.607564	newly 3 weeks cumul	122.	534.	{187., 865.}	337.705	0.549827	newly 5 weeks spec	8.	84.5614	{27., 128.}	957.018	0.870783	newly 5 weeks cumul	152.	727.333	{243., 1154.}	378.509	0.576587	newly 45 weeks cumul	158.	967.754	{303., 1498.}	512.503	0.668069
34	newly 3 weeks spec	95.	116.388	{52., 205.}	49.8818	0.18887	newly 3 weeks cumul	318.	484.857	{214., 802.}	65.8195	0.20483	newly 5 weeks spec	73.	67.0816	{30., 115.}	42.6614	0.205782	newly 5 weeks cumul	481.	640.306	{284., 1107.}	53.3752	0.183922	newly 45 weeks cumul	672.	839.163	{358., 1421.}	50.0456	0.182995
35	newly 3 weeks spec	44.	60.0484	{23., 100.}	72.0308	0.239567	newly 3 weeks cumul	154.	251.887	{87., 468.}	86.1437	0.240466	newly 5 weeks spec	28.	31.5645	{9., 57.}	63.1912	0.258627	newly 5 weeks cumul	217.	328.5	{112., 591.}	78.4228	0.234955	newly 45 weeks cumul	280.	410.597	{145., 734.}	76.0541	0.233906
36	newly 3 weeks spec	9.	58.8281	{20., 82.}	553.646	0.695456	newly 3 weeks cumul	71.	253.609	{97., 372.}	257.196	0.436234	newly 5 weeks spec	1.	28.2969	{8., 41.}	2729.69	1.29674	newly 5 weeks cumul	72.	321.844	{117., 457.}	347.005	0.533404	newly 47 weeks cumul	75.	389.859	{146., 555.}	419.813	0.595195
37	newly 3 weeks spec	26.	12.05	{0., 30.}	74.4231	0.840867	newly 3 weeks cumul	60.	65.925	{13., 144.}	85.4583	0.423782	newly 5 weeks spec	12.	4.3	{0., 14.}	80.8333	1.11276	newly 5 weeks cumul	86.	77.5	{14., 178.}	79.8256	0.477485	newly 50 weeks cumul	182.	82.75	{16., 183.}	71.7308	0.667104
38	newly 3 weeks spec	105.	62.902	{23., 152.}	56.6013	0.381933	newly 3 weeks cumul	339.	272.725	{112., 625.}	50.9688	0.27509	newly 5 weeks spec	72.	32.7843	{10., 67.}	59.695	0.464821	newly 5 weeks cumul	490.	352.176	{141., 809.}	51.5126	0.302968	newly 46 weeks cumul	831.	435.824	{176., 1010.}	57.1624	0.403237
39	newly 3 weeks spec	5.	26.3103	{8., 52.}	427.586	0.607918	newly 3 weeks cumul	20.	121.776	{42., 237.}	508.879	0.676647	newly 5 weeks spec	0.1	10.7241	{2., 24.}	10 627.6	1.85602	newly 5 weeks cumul	20.	150.621	{50., 288.}	653.103	0.766977	newly 47 weeks cumul	20.	171.483	{55., 361.}	757.414	0.820092
40	newly 3 weeks spec	69.	89.0667	{28., 164.}	66.9565	0.239905	newly 3 weeks cumul	225.	295.7	{102., 596.}	67.5704	0.239105	newly 5 weeks spec	51.	71.8	{19., 142.}	85.4902	0.284577	newly 5 weeks cumul	346.	444.	{136., 874.}	68.0539	0.24422	newly 20 weeks cumul	809.	924.267	{252., 1614.}	67.9604	0.273962
41	newly 3 weeks spec	0.1	4.06667	{0., 12.}	4002.22	1.25726	newly 3 weeks cumul	0.1	24.8222	{6., 61.}	24 726.7	2.20238	newly 5 weeks spec	0.1	1.11111	{0., 4.}	1140.	0.484188	newly 5 weeks cumul	0.1	28.1111	{6., 69.}	28 015.6	2.24472	newly 51 weeks cumul	0.1	29.0889	{6., 76.}	28 993.3	2.25414
42	newly 3 weeks spec	35.	67.4444	{21., 105.}	108.19	0.26438	newly 3 weeks cumul	180.	278.556	{102., 441.}	77.9383	0.223644	newly 5 weeks spec	44.	41.0667	{11., 65.}	58.9899	0.28984	newly 5 weeks cumul	253.	372.044	{133., 558.}	73.5617	0.220703	newly 43 weeks cumul	319.	487.156	{160., 727.}	77.4434	0.222904
43	newly 3 weeks spec	0.1	18.1951	{4., 30.}	18 095.1	2.0981	newly 3 weeks cumul	1.	92.5366	{38., 143.}	9153.66	1.8467	newly 5 weeks spec	0.1	7.2439	{1., 11.}	7153.66	1.64347	newly 5 weeks cumul	1.	111.951	{45., 175.}	11 095.1	1.92546	newly 49 weeks cumul	1.	123.317	{45., 203.}	12 231.7	1.95658
44	newly 3 weeks spec	90.	144.344	{46., 225.}	83.0783	0.243787	newly 3 weeks cumul	310.	574.459	{218., 868.}	98.0857	0.258577	newly 5 weeks spec	69.	81.459	{34., 123.}	58.1611	0.22019	newly 5 weeks cumul	468.	764.459	{292., 1147.}	81.1756	0.230752	newly 45 weeks cumul	766.	1008.79	{374., 1508.}	62.9457	0.2143
45	newly 3 weeks spec	7.	24.9091	{7., 51.}	260.	0.449014	newly 3 weeks cumul	18.	134.109	{40., 281.}	645.051	0.754082	newly 5 weeks spec	3.	8.01818	{1., 16.}	193.939	0.45886	newly 5 weeks cumul	26.	156.473	{45., 328.}	501.818	0.661573	newly 52 weeks cumul	26.	167.745	{49., 348.}	545.175	0.690711
46	newly 3 weeks spec	14.	45.05	{20., 84.}	225.357	0.438858	newly 3 weeks cumul	72.	215.075	{75., 371.}	199.896	0.407209	newly 5 weeks spec	4.	20.525	{6., 34.}	414.375	0.609087	newly 5 weeks cumul	77.	265.6	{99., 461.}	245.13	0.463321	newly 49 weeks cumul	79.	303.675	{132., 586.}	284.399	0.507873
47	newly 3 weeks spec	62.	102.745	{42., 183.}	83.2551	0.226411	newly 3 weeks cumul	217.	454.327	{177., 809.}	117.361	0.282978	newly 5 weeks spec	38.	51.0364	{19., 82.}	67.1292	0.230311	newly 5 weeks cumul	302.	575.382	{220., 1009.}	100.891	0.254626	newly 49 weeks cumul	355.	685.564	{261., 1205.}	103.872	0.260577
48	newly 3 weeks spec	10.	96.5882	{35., 154.}	865.882	0.866837	newly 3 weeks cumul	99.	403.686	{165., 666.}	308.239	0.511817	newly 5 weeks spec	18.	51.1176	{17., 82.}	191.83	0.350859	newly 5 weeks cumul	143.	527.902	{210., 863.}	270.04	0.465079	newly 46 weeks cumul	178.	651.	{251., 1061.}	266.413	0.454802
49	newly 3 weeks spec	135.	110.974	{29., 195.}	65.698	0.366942	newly 3 weeks cumul	399.	405.667	{123., 834.}	62.2968	0.286341	newly 5 weeks spec	100.	76.2821	{15., 155.}	77.2051	0.448453	newly 5 weeks cumul	612.	573.513	{168., 1179.}	63.3442	0.311926	newly 42 weeks cumul	1281.	878.077	{203., 1670.}	71.983	0.446376
50	newly 3 weeks spec	34.	33.325	{8., 50.}	65.5147	0.293366	newly 3 weeks cumul	108.	142.975	{45., 230.}	75.2546	0.239364	newly 5 weeks spec	11.	17.875	{3., 33.}	103.864	0.330176	newly 5 weeks cumul	144.	185.8	{68., 299.}	74.4444	0.242887	newly 42 weeks cumul	204.	233.775	{79., 414.}	71.973	0.269421
